# CO_2_-Optimization of Post-Tensioned Concrete Slab-Bridge Decks Using Surrogate Modeling

**DOI:** 10.3390/ma15144776

**Published:** 2022-07-07

**Authors:** Lorena Yepes-Bellver, Alejandro Brun-Izquierdo, Julián Alcalá, Víctor Yepes

**Affiliations:** 1 School of Civil Engineering, Universitat Politècnica de València, 46022 Valencia, Spain; lorena.yebe@gmail.com (L.Y.-B.); albruiz1994@gmail.com (A.B.-I.); 2 Institute of Concrete Science and Technology (ICITECH), Universitat Politècnica de València, 46022 Valencia, Spain; jualgon@cst.upv.es

**Keywords:** CO_2_ emission, optimization, metamodel, Kriging, post-tensioned concrete, structural optimization

## Abstract

This paper deals with optimizing embedded carbon dioxide (CO_2_) emissions using surrogate modeling, whether it is the deck of a post-tensioned cast-in-place concrete slab bridge or any other design structure. The main contribution of this proposal is that it allows optimizing structures methodically and sequentially. The approach presents two sequential phases of optimization, the first one of diversification and the second one of intensification of the search for optimums. Finally, with the amount of CO_2_ emissions and the differentiating characteristics of each design, a heuristic optimization based on a Kriging metamodel is performed. An optimized solution with lower emissions than the analyzed sample is obtained. If CO_2_ emissions were to be reduced, design recommendations would be to use slendernesses as high as possible, in the range of 1/30, which implies a more significant amount of passive reinforcement. This increase in passive reinforcement is compensated by reducing the measurement of concrete and active reinforcement. Another important conclusion is that reducing emissions is related to cost savings. Furthermore, it has been corroborated that for a cost increase of less than 1%, decreases in emissions emitted into the atmosphere of more than 2% can be achieved.

## 1. Introduction

The reduction of CO_2_ emissions is a relevant factor in the current climate change situation. The need to reduce the carbon footprint affects all human activities, including construction-related ones [1]. Indeed, construction contributes more than 40% of the world’s energy consumption and over one-third of greenhouse gas emissions [2]. It is estimated that the global production of structural concrete accounts for more than 5% of carbon emissions [3]. Therefore, there has been a progressive interest in incorporating environmental sustainability optimization in the construction sector [4].

Sustainable concrete structures can be optimized under different routes, including selecting construction materials with designs that require minimum energy or optimizing structural systems. One possibility is using new types of materials, such as infilled cementitious composites, which can significantly reduce the carbon footprint [5,6]. Another possibility is the use of recycled steel fibers [7]. Likewise, the optimization of concrete structures reduces carbon emissions and construction costs [8]. Heuristic optimization of CO_2_ emissions has been performed on building structures [9,10,11,12], bridge piers [13], or retaining walls [14,15,16], among other types of structures. In the cases studied, it is found that cost and emissions are related since they decrease as the material used is reduced.

Heuristic optimization of bridges, taking CO_2_ emissions as an objective function, has been the subject of several works by our research group. Yepes et al. [17] optimized a prefabricated trough girder bridge with a hybrid firefly-based algorithm, showing that a €1 reduction can save up to 1.75 kg of CO_2_. García-Segura et al. [18] optimize a post-tensioned box-section footbridge, whose results indicate that the emission reduction is achieved with more significant edges, more active reinforcement cables, and lower concrete characteristic strength. García-Segura and Yepes [19] performed a multi-objective optimization with cost, emissions, and safety for a post-tensioned box-section bridge. Penadés-Plà et al. [20] compared two box-section bridges showing that the emissions in the demolition phase are higher than those in the maintenance and repair phases. It was also shown that, although carbon emissions are an essential indicator of environmental impact, in some cases, it is insufficient, and other environmental impacts should be considered [21]. Yepes et al. [22] applied different metaheuristics to optimize cost and emissions in a single-span mixed concrete and steel footbridge. Martinez-Muñoz et al. [23] perform a recent review of research works related to composite bridges.

However, one of the limitations of heuristic optimization of structures is its high computational cost. Approximations or metamodels are often used. A metamodel replaces a simulation model to solve this problem. Kriging, Neural Networks (NNs), or Radial Basis Functions (RBFs) are among the most used metamodels in structural design optimization [24]. Although few works use it to design real-life structures, Kriging is one of the most promising surrogate models in structural optimization [25]. This model provides an optimal interpolation based on the regression of the observed values, weighted according to the spatial covariance values. Martínez-Frutos and Martí [26] use Kriging to solve the robust optimal design of articulated structures, decoupling the processes of uncertainty assessment from the optimization itself. This methodology was also recently applied to other fields such as wind turbine optimization [27] or railroad slab tracks [28].

In conjunction with heuristic optimization algorithms, Kriging has been used recently in bridge optimization, especially when using finite elements consumes much computational time [29,30]. In the specific field of the use of Kriging in box-section bridges, it was applied to help their multi-objective optimization subject to uncertainty [31]. An optimization algorithm based on Kriging reduced the computation time by 99.06% and obtained box-section bridges that differed by only 2.54% from those obtained with heuristic optimization by simulated annealing [32]. Penadés-Plà et al. [33] apply this metamodel to perform a prestressed box-section footbridge’s economic and robust optimization. Other recent works apply Kriging to optimizing light-weighted slab-bridges in CO_2_ emissions [34] and energy [35].

This article aims to design a methodology to reduce the carbon footprint in executing multiple voided slab decks for post-tensioned road flyovers. This study deals for the first time with optimization using a Kriging metamodel to optimize this post-tensioned concrete slab-bridge deck. The relevance of this approach lies in the fact that it allows us to tackle complex problems with a large number of variables or when the constraints have a long computational time. The proposed method has a general character, being able to be used in any other type of structure to optimize different objective functions. This work has two novel contributions: the application of a two-phase Kriging metamodel and the environmental optimization of lightened slab bridges.

## 2. Description of the Lightened Slab Bridge Deck

Designing a slab deck as a continuous hyperstatic beam with prestressed concrete is common. The slab solution is used for bridges with a principal span between 10 m and 45 m. They compete with precast girder bridges due to structural advantages (greater torsional and bending stiffness, greater durability and safety due to hyperstaticity) and constructive advantages since they adapt to difficult decks with formwork concreting simpler than in other typologies. Other advantages should be added, such as the elimination of roadway joints (increasing user comfort and reducing the risk of deck degradation), greater freedom to arrange support elements, and better aesthetic qualities. The aesthetics of the bridge is an essential factor when walking or visiting the structure underneath, which is favored by adapting the formwork to any section or curvature.

**Figure 1:**
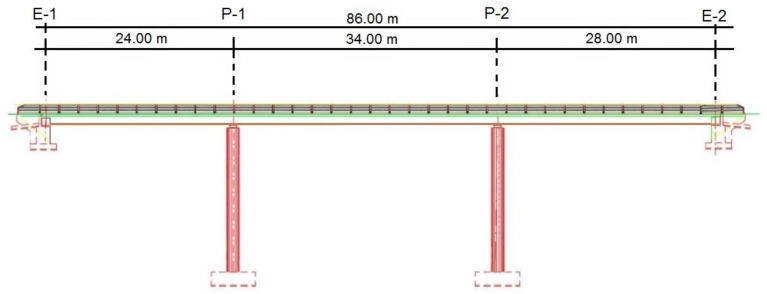
Prestressed concrete slab road bridge longitudinal profile.

**Figure 2:**
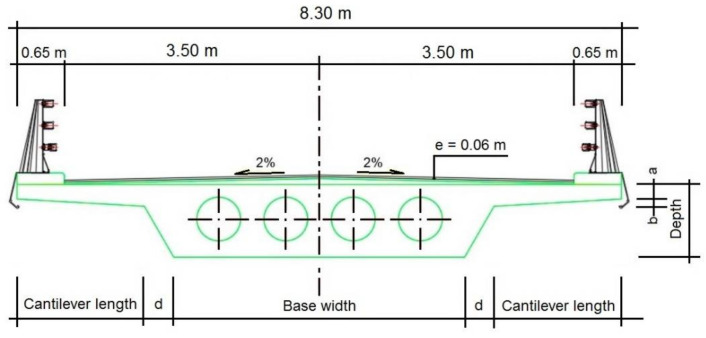
Typical light-weighted gull-wing section deck.

In this work, multiple voided slab decks for post-tensioned road flyovers with three spans of 24-34-28 m, with a total length of 86 m, will be optimized. It is resolved using an in-situ slab with constant depth and a straight profile (Figure 1). The deck has 8.30 m in width, composed of two 3.50 m lanes and barriers on both sides of 0.65 m each, together with a concrete pedestal. Figure 2 shows the characteristics and dimensions of the cross-section.

The limit state theory allows structures to be checked by partial safety coefficients. In each design situation, it is verified that no limit state is exceeded, considering both the ultimate and serviceability limit states.

The deck was calculated by the CSiBridge v.21.0.0 bridge modeling, analysis, and design program. The structural analysis of each alternative obtained the acting stresses, represented in the form of sectional forces derived from the mathematical design model on which the actions are applied. In addition, the stress-resultants of each element are obtained, represented as the stresses resisted by each section. The acting and resisting stresses are obtained separately, with a sectional conception of the design of each structural element. The details of the calculation of this structure can be found in [34].

## 3. Methodology

This paper proposes a procedure to reduce carbon emissions in executing post-tensioned road flyovers. The methodology deploys two consecutive optimization phases; the first one of diversification and the second one of intensification in the search for the optimum. A Latin hypercube sampling is performed in both phases, and each deck alternative is analyzed to obtain the CO_2_ emissions. A response surface generated by a Kriging metamodel is optimized with these values.

### 3.1. Sampling Method

Latin hypercube sampling (LHS) selects random numbers in which the samples are uniformly distributed in a sample space. It generates a random sample in each interval and for each variable and performs random linkage between variables to obtain a vector of input values for each interval.

**Figure 3:**
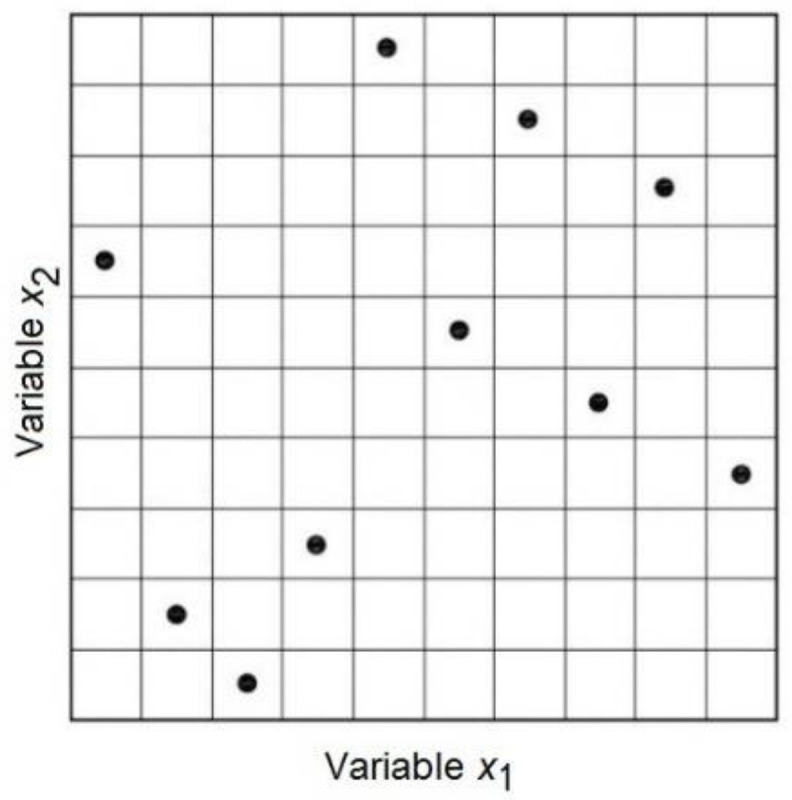
Latin hypercube sampling example for two variables and 10 sample points.

The numerical model is run as many times as intervals considered in the division of the probability distributions, independently of the number of variables sampled. Figure 3 shows an example of how the LHS works for the case of sampling with two design variables and ten samples.

Therefore, the number and location of the points determine the sampling. The sample size is increasingly related to the number of variables for the same accuracy of the metamodel. For concrete structures, sample sizes of around 30 individuals provide sufficiently good results [29,30,31,32].

The random values obtained ranged from 0 to 1, associated with each parameter to be modified once the LHS has been performed. The next step would be to fit the values into the ranges established for each variable. After this fitting, the values obtained will be adjusted for the variables within the established ranges, giving rise to the different solutions.

With this sampling, designs are obtained that conform the input to the optimization model after its structural analysis and comply with all the Limit States, being a bridge deck that can be executed.

### 3.2. CO_2_ Emissions Assessment

Each bridge deck has associated CO_2_ emissions. In order to compare the designs, the elements relevant to emissions were analyzed, in particular, the type of concrete (strength), the formwork surface used, the amount of steel, and the voids volume. This study analyzes sustainability based on a function of CO_2_ emissions during construction. To this end, the values for materials were taken from the BEDEC materials database [36]. The data do not reflect transportation emissions, which are highly dependent in all case studies. Note that concrete unit emissions were determined from each mix design. The emissions were evaluated according to the data shown in Table 1.

Emissions are associated with the measurements of each material, resulting in the amount of CO_2_. The direct emissions of the materials used are considered since they are the ones that make the difference between the alternatives.

At this point, a study of conventional solutions would conclude with the selection of the deck that produces the least amount of emissions. However, a methodology is proposed that, based on the set of alternatives resulting from the sampling, can reduce emissions by applying optimization on a predictive model such as Kriging.

### 3.3. Response Surface Optimization and Generation Method

Compared to traditional analysis, the proposed methodology presents a different approach. Based on the LHS sampling, a Kriging-type predictive metamodel is fitted that can be optimized using a heuristic algorithm.

Heuristic algorithms use artificial intelligence-based techniques to select a design, analyze the structure, control the constraints and redesign the structure by altering the variables until the objective function is optimized. Heuristic optimization is very effective because, although it does not guarantee obtaining the global optimum of the problem, it provides near-optimal solutions in reasonable computational times.

Usually, structural optimization is performed by heuristic optimization because of the high complexity of structural problems. However, standard heuristic optimization is still very time-consuming.

A practical solution to optimize with the lower computational cost is using approximate response surfaces generated by surrogate models or metamodels. The most common metamodels are polynomial regression, neural networks, and Kriging. These models are adjusted after evaluating a set of points in the solution space generated by sampling, obtaining an approximate response more quickly than using the real model.

Kriging-based heuristic optimization is proposed as different from standard heuristic optimization to perform accelerated optimization of complex problems [32]. Kriging makes available an optimal regression-based interpolation in contrast to the observed values of the neighboring data points, weighted according to the spatial covariance values. That is, the model takes into account global and local approximations simultaneously. Thus, Kriging considers the local variations of the objective response.

**Figure 4:**
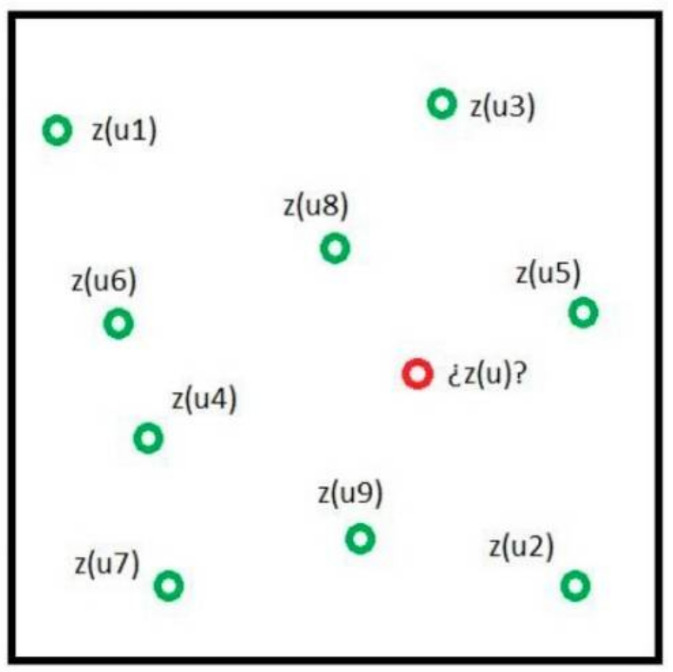
The basic idea of the Kriging predictive model.

The basic idea of Kriging is to predict the unknown value of an attribute *z* at coordinate point *u* from *n* known values of *z*, whose coordinates are *u_α_*, with α = 1, ..., *n*. Figure 4 expresses this idea. In this case, the attribute is the emissions produced by the execution of the deck, and the points are the set of solutions extracted by LHS sampling.

The rationale of metamodels is to assemble an approximate model of the objective function starting from a set of points in the solution space (initial sampling) to predict the data output without performing a complete structural analysis.

The construction of a metamodel is centralized in three parts: obtaining the initial input points within the design space, selecting the type of metamodel to build the approximate mathematical model, and choosing the connection pattern. The main objective in building a metamodel is to achieve the best possible accuracy in predicting the objective response.

**Figure 5:**
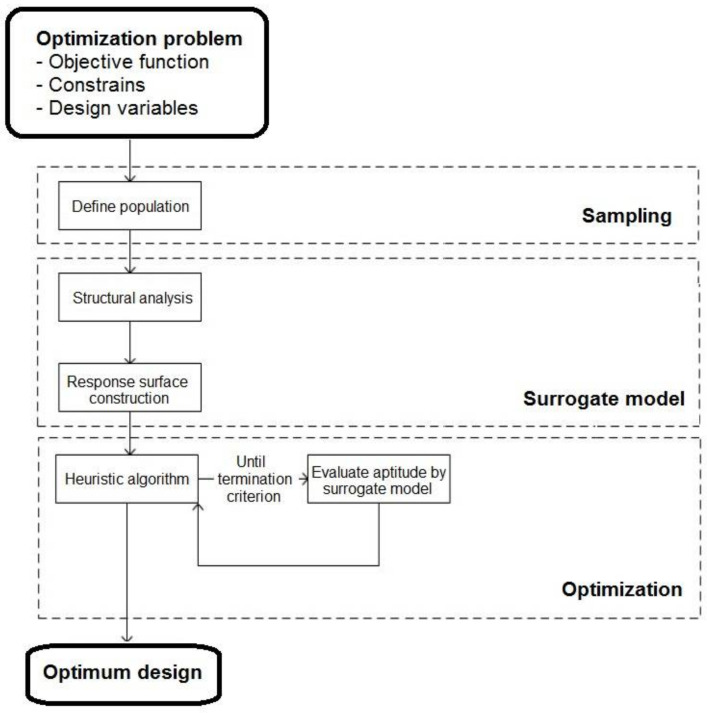
Metamodel-based heuristic optimization flowchart [32].

The most time-consuming part of the standard heuristic optimization process, the structural analysis, and evaluation of the objective function, is substituted by a metamodel prediction. Consequently, the computational cost required for surrogate model-based heuristic optimization, as seen in Figure 5, is lower than the computational cost required for standard heuristic optimization [32].

Kriging-based optimization offers results close to standard heuristic optimization using less time [32]. Therefore, this model solves structures that involve a very high computational cost while reducing the difficulty of other problems.

For this purpose, the “MATLAB Kriging Toolbox” (DACE, Design, and Analysis of Computer Experiments) has been used. This is a toolbox for working with Kriging approximations [37]. The usual use of this software is to construct a Kriging model based on data from a computer experiment and use it as a surrogate for the original one. Here, a computer experiment is a collection of input-response pairs of model runs.

**Figure 6:**
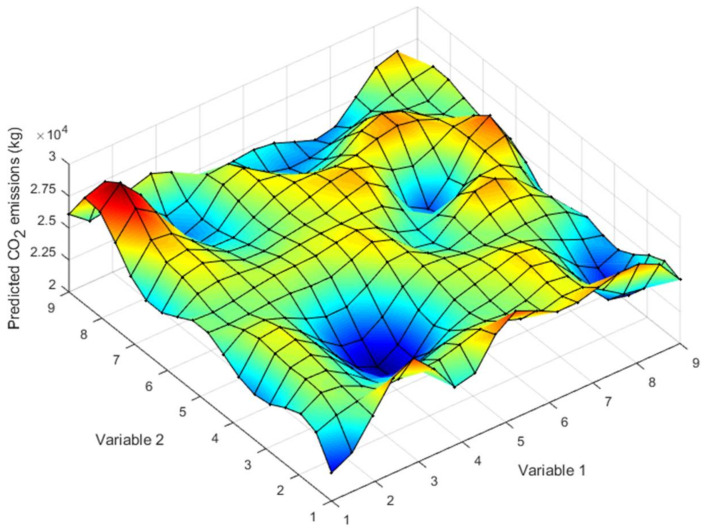
Example of response surface.

The purpose is to obtain the response surface for optimization. Figure 6 shows an example proposed in that paper, where from a series of points in space, a correlation between them is obtained. The document DACE explains all the mathematical apparatus, a MATLAB Kriging Toolbox, Version 2.0 [37].

The heuristic algorithm used is simulated annealing [38]. Its name is because the optimization process simulates the process by which the crystal molecules reach a minimum energy state. The acceptance of a new solution is governed by the probabilistic expression exp(−Δ*E*/*T*), where Δ*E* is the energy increment of a new configuration (difference between the values of the objective function of the candidate solution and the current one), and *T* is the temperature. Therefore, this method admits solutions that worsen the objective function and thus escape from local optima to find the global optimum. The initial temperature *T*_0_ is set following Medina’s method [39], which halves the temperature when the acceptance ratio exceeds 40% and doubles it when the ratio is less than 20%. The temperature is reduced geometrically by the expression *T* = *kT* each time it lasts 1000 Markov chains, according to a cooling coefficient *k* = 0.8. This temperature reduction reduces the probability of accepting a worse solution. Other authors use this algorithm to optimize structures, given its good convergence to the global optimum [40].

## 4. Results

### 4.1. Diversification Phase

In the first phase, LHS sampling is performed to diversify the search for the local optimum. The variables are the strength of the concrete in simple compression and the depth and base concerning the slab cross-section geometry. The remaining dimensions and voids are determined according to the relationships described in Table 2. The nomenclature of the variables corresponds to Figure 2. Therefore, the characteristic resistance of the concrete in simple compression is modified, taking values from 30 to 50 MPa. In addition, the depth will be varied, in a range from 1.15 m to 1.70 m, increasing the designs obtained by 5 cm, as well as the base of the section, which will take values from 3.00 m to 5.00 m, increasing, in the same way, each of the designs by 5 cm. The number of voids is the maximum number that can be fitted in the section. This explains why there are no void variables in Table 2. The minimum coating is the minimum distance between the void and the face of the deck or between the voids (void spacing).

Once the design variables have been determined, a sample is obtained by LHS to establish the different combinations of variables that will form part of the metamodel. The values obtained ranged from 0 to 1 (Table 3).

However, it has been assumed that the dimensions of both the edge and the bottom base would be within multiples of 5 cm. In addition, the values of the simple compressive strength of the concrete only take integer multiples of five. Therefore, the final dimensions that will give rise to the different panel solutions to be analyzed are shown in Table 4.

This sampling determines the data that feeds the Kriging model. The panels are analyzed and tested for both ultimate and serviceability limit states, and the emissions are quantified. The elements relevant to the amount of carbon are evaluated to compare the panels: the type of concrete used, the formwork area required, the amount of passive and active steel, and the voids volume. Emissions are related to the measurements of each material, resulting in kilograms of CO_2_. The direct emissions of each material are considered since they are the ones that make the difference in the volume of CO_2_ generated between the alternatives (Table 5).

A conventional solution study would conclude by selecting deck #30, which produces the least CO_2_. However, optimization on a Kriging model is proposed to obtain a solution with a lower carbon footprint than the sample analyzed. Once the response surface has been optimized with the simulated annealing, the characteristics of the optimum deck would be as shown in Table 6.

As can be seen, the optimized deck in the diversification phase reduces the percentage value of all the decks sampled, obtaining a reduction of up to 29% in the worst case and 1% in the best case. To calculate the relative reduction in emissions, the difference between the corresponding sample value and the optimum obtained has been divided by the optimum obtained.

### 4.2. Intensification Phase

The second phase intensifies the search for optimal solutions around the best solution obtained in the previous phase. First, sampling will be performed in the space close to the optimal solution of the diversification phase. As argued before, the design variables are the strength of the concrete in simple compression and the depth and base concerning the cross-section of the different prestressed slab decks to be analyzed. These variables have been reduced around the optimal diversification solution at this stage. The remaining dimensions and the number of cross-section lightenings are determined by the relationships described in Table 2.

Therefore, for the intensification, the concrete grade is modified from 35 to 45 MPa. In addition, as regards the geometry of the cross-section, the depth will be varied from 1.05 m to 1.25 m, increasing the designs obtained by 5 cm, as well as the base of the section, which will take values from 3.20 m to 3.90 m, increasing, likewise, by 5 cm.

Once the variables have been determined, the LHS sampling establishes the different combinations of variables that form part of the metamodel. It was decided to add five more individuals around the best diversification solution to corroborate whether this is a sufficient number of individuals to improve the solution obtained. Once the new decks have been analyzed, the restrictions required by the regulations are checked, and the emissions are evaluated (Table 7).

These new individuals do not improve the best solution found in the diversification phase. However, once the new response surface has been optimized, a different optimum improves the previous one (Table 8). Comparing the result obtained for emissions after optimizing this second phase, a decrease of up to 1.26% is obtained, concerning diversification, and a reduction of even 30.34% in the worst case. As was done for the diversification phase, the difference between the value of the corresponding sample and the optimum obtained was divided to estimate the relative reduction in emissions.

## 5. Discussion

Next, the results are discussed to draw practical conclusions from the bridge optimized for emissions (BOE, for simplicity) that can be used to design this structure. The BOE depth/span ratio is 1/30.91, slenderness at the limit recommended by the Dirección General de Carreteras [41], which advises slenderness between 1/22 and 1/30 for post-tensioned road flyovers. However, if we follow the suggestions of SETRA [42] for slabs with three or more spans and wide overhangs, its recommendation is 1/28. In both cases, the BOE tries to reduce the deck depth. If we compare this depth/span ratio with the statistical data from Yepes et al. [43], we see that the slenderness is greater than the 75% percentile, 1/26.39; moreover, only one of the cases analyzed showed slenderness greater than 1/30, which confirms that this optimum exhibits large slendernesses.

On the other hand, the BOE presents a concrete quantity of 0.56 m^3^ per m^2^ of the deck. This value is at the lower limit of the recommendations, which advise figures between 0.55 and 0.70 m^3^/m^2^ [41]. Moreover, it coincides with the 25% percentile of the sample [43]. The result confirms that the BOE aims to reduce the measurement of concrete. The amount of active prestressing for the BOE, 16.48 kg/m^2^ of the slab, although between the suggested limits [41], between 10 and 25 kg/m^2^, is below the 25% percentile [43]. This also indicates a tendency to lower the amount of active prestressing used.

The BOE passive reinforcement is 136.85 kg/m^3^ of concrete, which exceeds the suggestions, which advise figures between 70 and 100 kg/m^3^ [41]. However, the reality indicates that the median is 100.87 kg/m^3^ [43], implying that the recommendations [41] are below what is executed in real-life bridges. Moreover, the passive reinforcement of the best bridge is lower than the sample’s maximum, which was 187.08 kg/m^3^. If we analyze the BOE ratio of 77.00 kg/m^2^ of the deck, the figure is still high, exceeding the 75% percentile of the sample, although not reaching the maximum of 92.91 kg/m^2^. These figures conclude that the lower-emission decks prefer more passive reinforcement to reduce concrete and active prestressing.

Next, the BOE is compared to the predicted values [43]. This implies knowing whether the carbon footprint optimization involves changes concerning the linear models [41]. Indeed, in that work, knowing the main span and the internal and external lightening, the required active prestressing, the deck depth, and the concrete measurement could be predicted. In the case of the lower-emission bridge, the estimated active prestressing would be 21.22 kg/m^2^. However, the reality is that only 16.48 kg/m^2^ was required. Therefore, the BOE uses a smaller amount of active prestressing than is usually used. The design recommendation would be to reduce this amount as much as possible. Likewise, the estimated depth would be 1.13 m, while in BOE, it is 1.10 m, which is sensibly the same. The estimated concrete volume is 0.52 m^3^/m^2^, similar to the 0.56 m^3^/m^2^ of the BOE. These recommendations are consistent with those obtained by previous works [44] using heuristic optimization techniques, although in that case, with the objective function cost.

**Figure 7:**
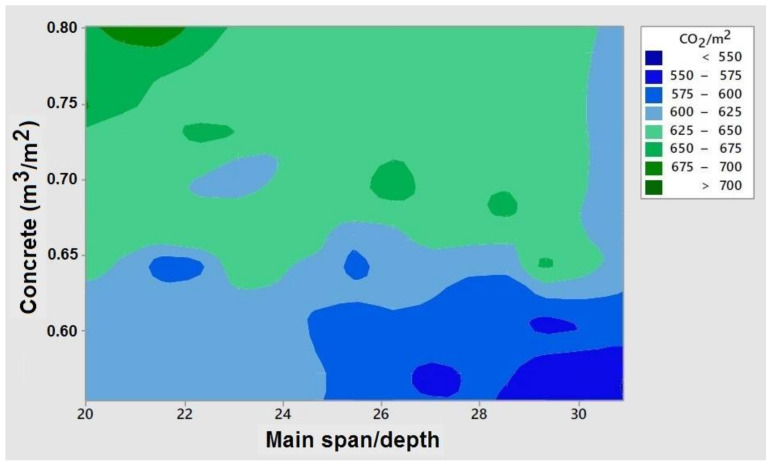
Contour plot of CO_2_/m^2^ with respect to main span/ridge and concrete amount.

Another relevant aspect is to check the dependence between the depth/main-span ratio and the concrete measurement. Figure 7 shows that high slendernesses, greater than 1/28, and concrete volumes as low as possible, less than 0.60 m^3^/m^2^, are interesting. The measurement of concrete obtained is consistent with the work of Alcalá [36], who, for the economic optimization of this type of bridge, proposes magnitudes of about 0.50 m^3^/m^2^. The same occurs with the depth/span ratio, around 1/25, with economic decks of larger spans.

On the other hand, we will compare what differences could be established between the economic cost of the bridge decks analyzed compared to the reduction of emissions. Table 9 shows the unit costs according to the type of material. It should be noted that only those costs that are comparable and relevant for comparison with CO_2_ emissions have been analyzed and contrasted [45]. By analyzing the cost of the decks analyzed, it can be seen that BOE costs 181,531 €, while Deck #30, the lowest cost of all, is 179,756 €. Therefore, BOE is 1% more expensive than Deck #30 but emits the least CO_2_. However, Deck #30 emits 2% more than the BOE, although it is the cheapest.

**Figure 8:**
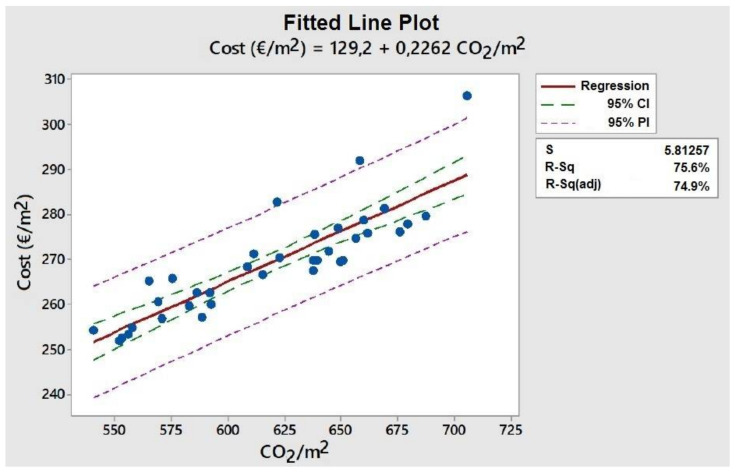
Fitted line plot between the emissions and the cost of the calculated bridge decks is referred to as the unit of deck area.

Figure 8 shows the line fitted to a linear model, where the cost of the bridge can be estimated as a function of emissions. Note that this linear model considers the common materials identified in Table 1. It is clear that by using other types of materials, including more recycled materials, the carbon footprint impact could be reduced. The meaning of the content of Figure 8 is as follows: S is the residual standard deviation; 95% CI is the confidence interval for the fit that provides a range of likely values for the mean response given the specified settings of the predictors; 95% PI is the prediction interval is a range that is likely to contain a single future response for a value of the predictor variable; R-sq is the percentage of variation in the response that the model explains; R-sq (adj) is the adjusted R-sq. It can be seen that the variability explained by the model is high, around 75%. However, the point corresponding to the lowest emission obtained from the calculated bridge decks is found to have a cost 1.05% higher than that predicted by the model (251.69 €/m^2^), although within the 95% confidence interval for the mean. The model tells us that, removing the fixed costs of 129.2 €/m^2^, for each kg of CO_2_/m^2^ that we reduce in our design, we will save 0.2262 €/m^2^.

## 6. Practical Recommendations

Practical recommendations can be offered for designing this structure to reduce CO_2_ emissions, considering the results obtained during the optimization process. The design recommendations are to use slendernesses as high as possible in the region of 1/30, which implies a more significant amount of passive reinforcement. This increase in passive reinforcement is compensated by reducing the measurement of concrete and active reinforcement. In turn, to reduce the volume of concrete. It is recommended to increase the lightening as much as possible to reduce the volume of concrete.

On a practical level, to reduce emissions in the case of a prestressed slab-bridge with three spans and a main span of 34 m, the following is recommended:

- Depth/span ratio greater than 1/28.
- Concrete measurements less than 0.60 m^3^/m^2^ of the deck.
- Amounts of passive reinforcement above 120 kg/m^3^ of concrete.
- Amounts of active prestressing below 17 kg/m^2^ of the deck.
- Concrete grade between C-35 and 40 MPa.
- External lightening between 0.40 and 0.50 m^3^/m^2^ of the deck.
- Interior lightening below 0.20 m^3^/m^2^ of the deck.

## 7. Conclusions

This paper presents a metamodel-based structure optimization methodology that has solved the problem in an agile and systematic way. It has been shown that the two-phase approach based on a Kriging metamodel improves the environmental design of a prestressed slab-bridge deck to mitigate CO_2_ emissions. It is also found that the reduction of CO_2_ is directly related to the cost. Cost optimization would therefore be sufficient to reduce the environmental aspects. In addition, it has been corroborated that a cost increase of less than 1 % leads to a reduction of more than 2 % in the amount of CO_2_ emissions released into the atmosphere. However, barring a drastic change in technology, the CO_2_ emissions of individual building units are more stable values than price fluctuations. A clear line of future research to apply this methodology to the entire life cycle analysis and its application to other objective functions such as constructability, safety, energy consumption, or others. In addition, the sensitivity of the parameters to the final results should also be studied.

## Figures and Tables

**Table 1:** CO_2_ emissions [36].

Material	kg CO_2_/kg	kg CO_2_/m^3^	kg CO_2_/m^2^
C-30 concrete		227.01	
C-35 concrete		263.96	
C-40 concrete		298.57	
C-45 concrete		330.25	
C-50 concrete		358.97	
Steel reinforcement	3.03		
Steel prestressed	5.64		
Formwork			2.24
Lightening		604.42	

**Table 2:** Ranges of dimensions and their limitations established by regulations [41].

Design Variables	Range	Limitation
Concrete grade (*f_ck_*)	30–50 MPa	-
Depth of the deck (*c*)	1.15–1.70 m	>0.90 m
Base width (*b*)	3.00–5.00 m	-
Cantilever length (*v*)	Variable	<3.50 m
Distance between cantilever and nucleus (*d*)	0.40 m	-
Cantilever starting thickness *e*_1_ (*a*+*b*)	0.35 m	-
Cantilever edge thickness *e*_2_ (*a*)	0.25 m	>0.20 m
Minimum void coating	0.225 m	>0.15 m

**Table 3:** Values obtained after Latin hypercube sampling.

Depth of the Deck (m)	Base Width (m)	Concrete Grade (MPa)	Depth of the Deck (m)	Base Width (m)	Concrete Grade (MPa)
0.58	0.61	0.27	0.30	0.96	0.38
0.74	0.48	0.31	0.93	0.216	0.24
0.52	0.86	0.17	0.88	0.096	0.63
0.99	0.28	0.79	0.19	0.11	0.74
0.10	0.31	0.59	0.49	0.00	0.45
0.76	0.18	0.83	0.58	0.97	0.64
0.14	0.91	0.91	0.37	0.61	0.17
0.03	0.71	0.96	0.85	0.36	0.74
0.42	0.38	0.11	0.58	0.78	0.73
0.33	0.69	0.08	0.72	0.21	0.08
0.36	0.56	0.67	0.18	0.00	0.99
0.63	0.75	0.05	0.48	0.46	0.87
0.82	0.53	0.49	0.68	0.59	0.37
0.21	0.82	0.54	0.98	0.87	0.56
0.68	0.43	0.87	0.06	0.76	0.45

**Table 4:** Values obtained for the design variables within established ranges.

Deck	Depth of the Deck (m)	Base Width (m)	Concrete Grade (MPa)	Deck	Depth of the Deck (m)	Base Width (m)	Concrete Grade (MPa)
1	1.45	4.35	35	16	1.30	4.90	40
2	1.55	4.10	35	17	1.65	3.65	35
3	1.45	4.75	35	18	1.65	3.45	45
4	1.70	3.80	45	19	1.25	3.50	45
5	1.20	3.85	40	20	1.40	3.30	40
6	1.55	3.60	45	21	1.45	3.90	45
7	1.20	4.85	50	22	1.35	3.60	35
8	1.15	4.50	50	23	1.50	3.35	45
9	1.35	3.95	30	24	1.50	4.50	45
10	1.30	4.45	30	25	1.55	3.20	30
11	1.35	4.25	45	26	1.25	3.00	50
12	1.50	4.55	30	27	1.40	3.45	45
13	1.60	4.20	40	28	1.50	3.55	35
14	1.25	4.70	40	29	1.70	3.85	40
15	1.50	4.05	45	30	1.15	3.70	40

**Table 5:** CO_2_ emissions of each of the decks analyzed.

CO_2_ (kg)	Deck	CO_2_ (kg)	Deck	CO_2_ (kg)
439,416	11	455,374	21	464,536
460,393	12	434,674	22	416,584
455,722	13	503,797	23	455,442
484,897	14	462,915	24	490,669
407,988	15	482,659	25	403,972
456,668	16	477,491	26	423,112
472,401	17	444,714	27	470,008
471,362	18	464,051	28	418,839
406,654	19	420,514	29	468,898
436,703	20	443,840	30	394,616

**Table 6:** Result after optimization of the diversification phase.

Depth of the Deck (m)	Base Width (m)	Concrete Grade (MPa)	CO_2_ (kg)
1.15	3.55	40	391,370.00

**Table 7:** Values obtained for the design variables within established ranges.

Deck	Depth of the Deck (m)	Base Width (m)	Concrete Grade (MPa)	CO_2_ (kg)
31	1.15	3.40	35	411,077.14
32	1.25	3.35	35	398,614.42
33	1.15	3.65	45	422,934.14
34	1.15	3.35	40	395,465.24
35	1.15	3.25	40	397,153.86

**Table 8:** Result after optimization of the intensification phase.

Depth of the Deck (m)	Base Width (m)	Concrete Grade (MPa)	CO_2_ (kg)
1.10	3.40	35	386,514.57

**Table 9:** Unit prices of materials [36].

Material	€/kg	€/m^3^	€/m^2^
C-30 concrete		99.81	
C-35 concrete		104.57	
C-40 concrete		109.33	
C-45 concrete		114.10	
C-50 concrete		118.87	
Steel reinforcement	1.16		
Steel prestressed	3.40		
Formwork			33.81
Lightweighting		99.81	

## Data Availability

Not applicable.
